# Gamma radiation shielding effectiveness of PbO_2_ doped borosilicate glasses

**DOI:** 10.1038/s41598-025-05275-8

**Published:** 2025-06-27

**Authors:** Mohamed Elsafi, Mohamed Y. Hanfi, Shoaa M. Al-Balawi, M. I. Sayyed

**Affiliations:** 1 Physics Department, Faculty of Science, Alexandria University, Alexandria, 21511 Egypt; 2 Ural Federal University, Ekaterinburg, 620002 Russia; 3 Nuclear Materials Authority, P.O. Box 530, El-Maadi, Cairo, Egypt; 4 General Science Program-Deanship of Support Studies, Alasala University, Dammam, Saudi Arabia; 5 Department of Physics, Faculty of Science, Isra University, Amman, Jordan

**Keywords:** Borosilicate glasses, Transmission factor, Mean free path, Gamma sources, Semiconductor detector, Physics, Nuclear physics

## Abstract

In this study, the integration of PbO_2_ into a borosilicate glass system was investigated for enhanced radiation shielding performance. Several glasses with varying PbO_2_ concentrations (31, 33, 35 and 37 mol%) were prepared using the melt-quenching method. The density of the glasses increases from 4.579 to 5.044 g/cm^3^ as a result of increase the PbO_2_ content. The radiation attenuation factors were experimentally determined at 0.059, 0.662, 1.173 and 1.333 MeV, using HPGe detector. The results indicate that increasing PbO_2_ content notably influences the mass attenuation coefficient and the effective atomic number. The tenth value layer (TVL) increased significantly with rising energy levels. For the glass sample containing 31 mol% PbO₂, the TVL increased from 0.177 cm at 0.059 MeV to 5.325 cm at 0.662 MeV, and to 9.094 cm at 1.333 MeV. Similarly, for the glass with 37 mol% PbO₂, the TVL increased from 0.146 cm at 0.059 MeV to 4.733 cm at 0.662 MeV, and to 8.231 cm at 1.333 MeV. The results also showed that PbO₂ has an inverse effect on the TVL, where adding more PbO₂ leads to a decrease in the TVL. At 0.662 MeV, increasing the PbO₂ content from 31 to 37 mol% reduces the TVL by approximately 11.12%. The transmission factor (TF) for the glass with a thickness of 2 cm was investigated, and results showed that the TF is nearly 0 at 0.059 MeV, indicating that the glass provides complete shielding at this low energy. The TF increases with rising energy, reaching 37.8–42.11% at 0.662 MeV, indicating that more photons penetrate the glass as the energy increases.

## Introduction

Concerning normal background radioactivity, mining, milling, artificial radio-isotope-powered generating stations, space flight, experimentation, and many other factors, humans are now exposed to ionizing radiation because of the exponential growth of technology. Radiation may outcome several medical conditions, with tissue damage, genetic alterations, and carcinomas being among those that are most serious. Moreover, ionizing radiation can change the chemical composition and structure of rock, water, and soil. It can also negatively impact biodiversity and the natural environment. Given their high level of danger to humans, serious thought is required, and effective shielding and protection methods are required^1–8^.

One of the best techniques to protect living things from the harmful effects of radiation exposure is to apply manufactured materials that are capable of attenuating the ionizing radiation, such as polymers, concrete, alloys, ceramics, and glass^5,9–16^. In order to protect biological tissue against ionizing radiation, the right materials must be used. Compared to traditional radiation protection materials, glass offers many benefits. It is capable of efficiently blocking ionizing irradiation, notably when it is doped with high atomic number compounds, PbO_2_ and SiO_2_. Glass does not readily break down; it is resistant to corrosion and chemical materials. It has a changeable density and composition for optimal shielding effectiveness, and it is mechanically durable. Glass is capable of being formed into a wide range of shapes and sizes, unlike traditional materials^6,17–22^.

The “shielding efficiency” is based on the stopping power of the shielding medium. It relies on the strength and intensity of the radiation. Another important factor is the shielding material. Lead and Pb-equivalent materials have most widely been utilized for radiation protection. When the composition of glass contains lead oxide (PbO_2_), its radiation shielding ability becomes much better. Lead oxide increases the density and atomic number as well as the shielding characteristics of glass material against ionizing radiation. Glass that contains PbO_2_ is transparently clear and can be visually monitored in radiation environments. This is unlike lead sheets which is opaque. Chemical durability, mechanical stability and long-lasting are all satisfactory. When PbO_2_ is added it raises refractive index and optical properties to make it favourable for some applications such as radiation shielding window /lead glass and protective Silicon dioxide participates mainly in glass compositions^23–28^. It affects the properties, structure, and radiation protection of any material. SiO₂ is an important glass forming. It develops a strong and durable network that offers chemical reactivity, mechanical strength, and resistance to degradation from the environment. On the contrary, pure silica glass has a high melting point and inefficient reliability. To improve fluidity, reduce melting point, and increase industrial application on the glass maker’s part, sodium oxide (Na_2_O) is added to the silica and acts as a network-modifier, by opening some of the Si-O links. The SiO₂-Na₂O composition provides a durable matrix for the dispersion of heavy metal oxides, like PbO_2_, resulting in radiation attenuation and eventual radiation shielding uniformity. The glass is capable of providing an evenly distributed and amorphous structure that maximizes the glass’s ability to screen ionizing radiation and maintain a high optical quality^29–35^.

Using a borate-based glass network, we integrated the three interesting oxides (Na_2_O, PbO_2_, and SiO_2_) and examined the impact of substituting PbO_2_ and SiO_2_ with B_2_O_3_ on the shielding properties of the suggested glass matrix.

## Materials and methods

**Fig. 1:**
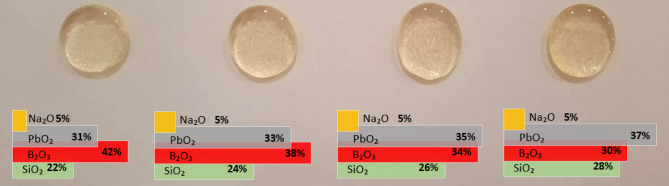
The photo for the prepared glasses.

Sodium lead borosilicate glasses were fabricated by the melt quenching approach. The following oxides were the raw materials to fabricate the current samples: Na_2_O, PbO_2_, SiO_2_ and B_2_O_3_. Weighed raw powders were mixed for few minutes in an alumina crucible. A batch of 20 g powders mixture was subsequently heated to 1100 °C for 60–75 min in a muffle furnace. The purpose of this step is to obtain homogeneous melt free of bubbles. In order to get the samples presented in Fig. 1, the melt was poured into stainless steel plate. The resultant glass samples were annealed for 4 h in a furnace to remove the internal stress. Table 1 summarizes the amount (in mol%) of each oxide in the present glasses. The exact densities of the four glass samples are calculated using Archimedes’ formula as it is a very important parameter in our study. The (M_L_) and (M_r_) numbers are representations of the weight of the glass in liquid and air. If water is used in this case as the immersing liquid, the density of the liquid value is given by the ρ_L_ value and is taken as 1 g/cm^3^^36–38^.

1 $$\:{\rho\:}_{g}=\frac{{M}_{r}}{{M}_{r}\text{}\text{\--}\text{}{M}_{L}}{\rho\:}_{L}$$

**Table 1:** The chemical composition of NPBS glasses and their corresponding density (g/cm^3^) .

Sample code	Comp, mol%				Density, g cm^-3^
	Na_2_O	PbO_2_	B_2_O_3_	SiO_2_	
NPBS-1	5	31	42	22	4.579
NPBS-2	5	33	38	24	4.732
NPBS-3	5	35	34	26	4.887
NPBS-4	5	37	30	28	5.044

**Fig. 2:**
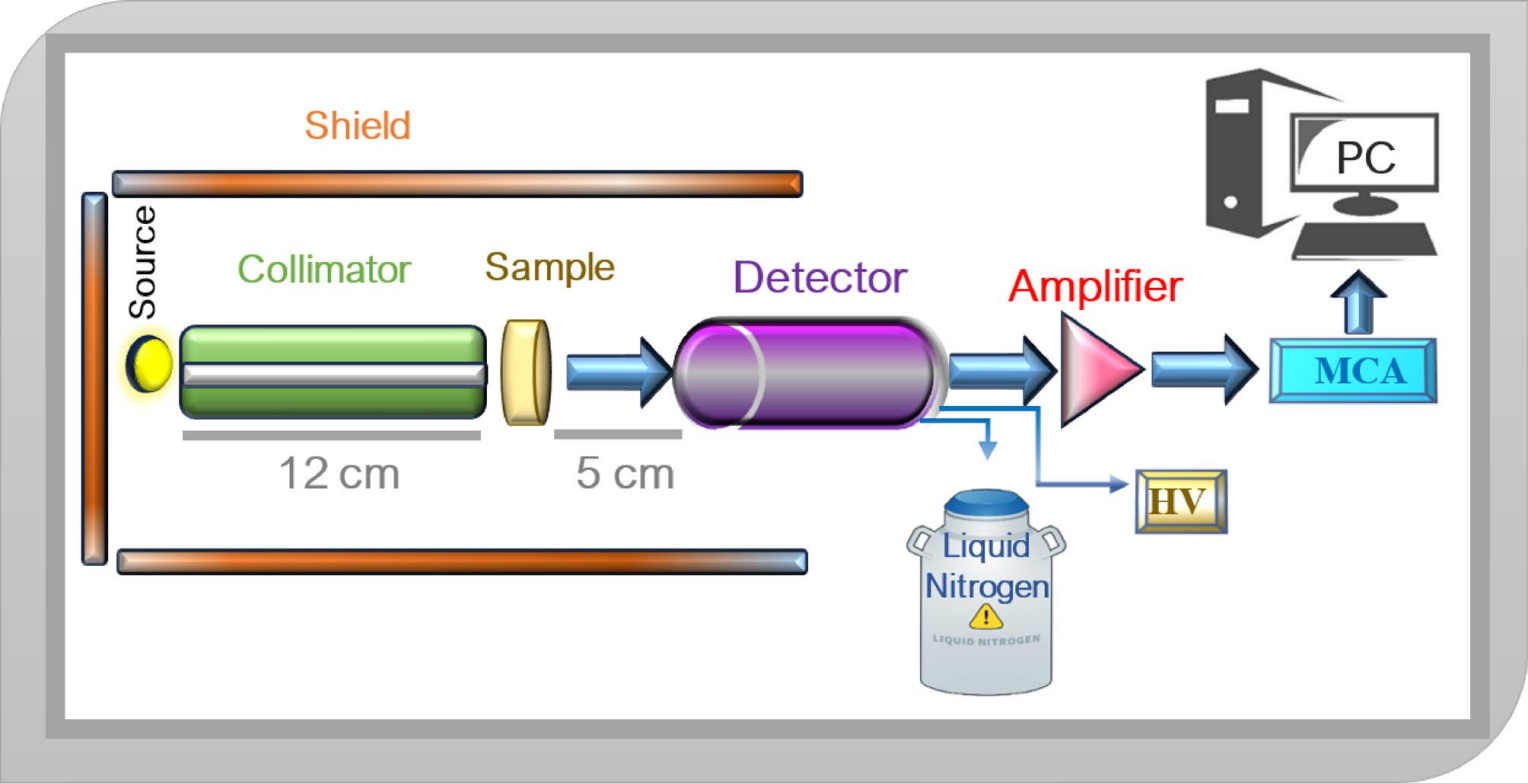
The experimental work geometry.

The experimental shielding characteristics of the manufactured glass samples were tested using an HPGe detector^39^ against the four energy ranges of three sources Am-241, Co-60, and Cs-137. The geometry of the configuration used in this study, shown in Fig. 2, used the narrow beam method. A glass sample was placed as shown in Fig. 2 between a source of specific energy photons and the detector. The transmission intensity after sufficient measured time (to give error less than 1%) can be measured by knowing the area under the photopeak (A), the measuring without the glass absorber at the same time give the initial intensity or (A_O_). By calculating the thickness of glass (t) as well as the measured values A and A_O_, The LAC can be estimated by the next formula^5,40–46^.

2 $$\:LAC=\frac{1}{x}\text{ln}\frac{{A}_{0}}{A\:}$$

Related essential parameters such as half value layer (HVL), MFP (mean free path), tenth value layer (TVL) and RSE (Radiation shielding efficiency) were estimated by the following Eqs.^6,47–53^.

3 $$\:HVL=\frac{Ln\:\left(2\right)}{LAC}$$

4 $$\:MFP=\frac{1}{LAC}$$

5 $$\:TVL=\frac{Ln\:\left(10\right)}{LAC}$$

6 $$\:RPE,\:\%=[1-\frac{A}{{A}_{0}\:}]\times\:100$$.

## Results and discussion

**Fig. 3:**
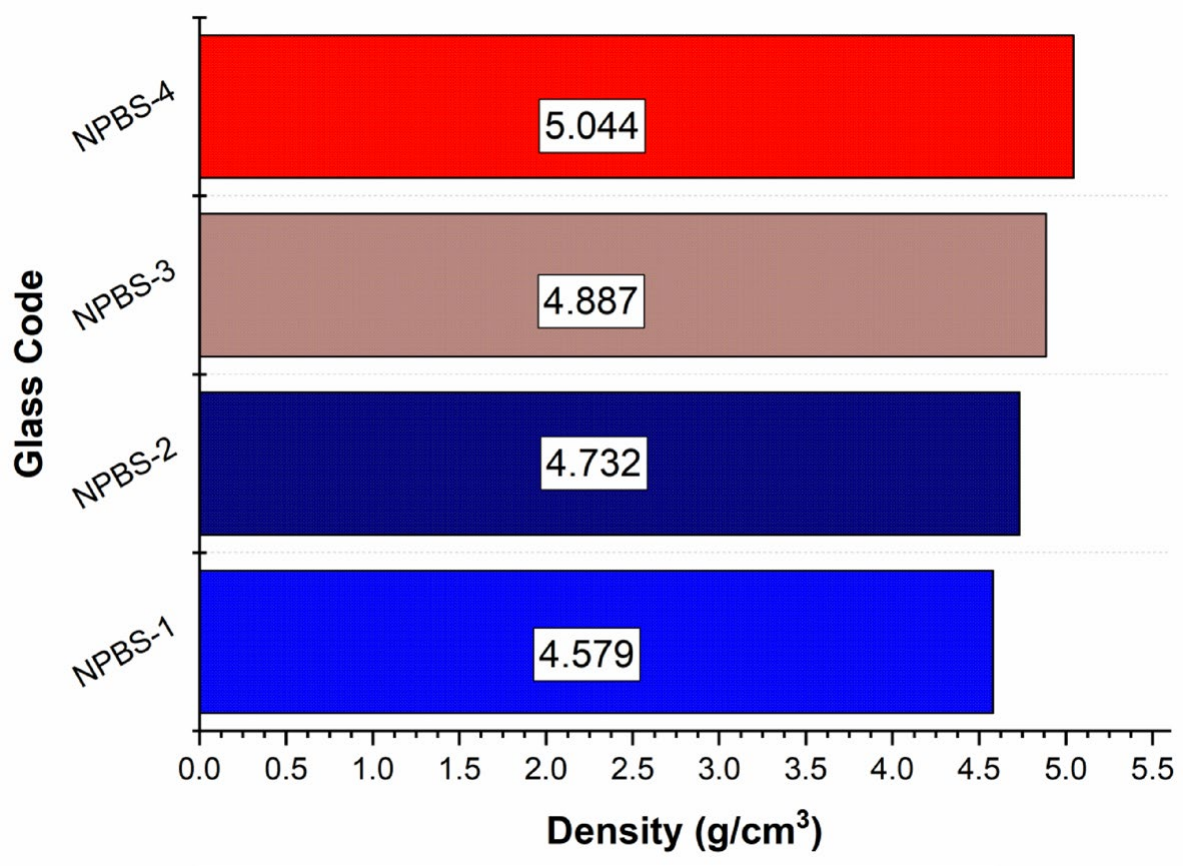
The Influence of variation of PbO and B_2_O_3_ concentration in NPBS glasses.

The density of NPBS glass is measured and presented in Fig. 3. The density values of NPBS glass samples are systematically rising, with NPBS-1 having the lowest density (4.579 g/cm^3^) and NPBS-4 having the highest density (5.044 g/cm^3^). This trend is primarily due to the increased content of PbO_2_, which clearly affects the cohesion of the glass structure. The high atomic mass of PbO_2_ in the composition of NPBS glass enhances the density, while the lattice components are affected by B_2_O_3_ and SiO_2_, which contributes to structural stability. Moreover, sodium oxide (Na_2_O) acts as a modifier, affecting the glass Lattice and overall density. The interaction of these oxides determines the physical properties of NPBS glass. Higher densities correspond to more compact atomic arrangements, which can enhance the glass’s shielding capabilities against ionizing radiation. The differences observed between NPBS-2 (4.732 g/cm^3^) and NPBS-3 (4.887 g/cm^3^) indicate how slight compositional changes affect the density. These findings are advantageous for applications requiring high-density glass materials, such as radiation shielding and optical components.

**Fig. 4:**
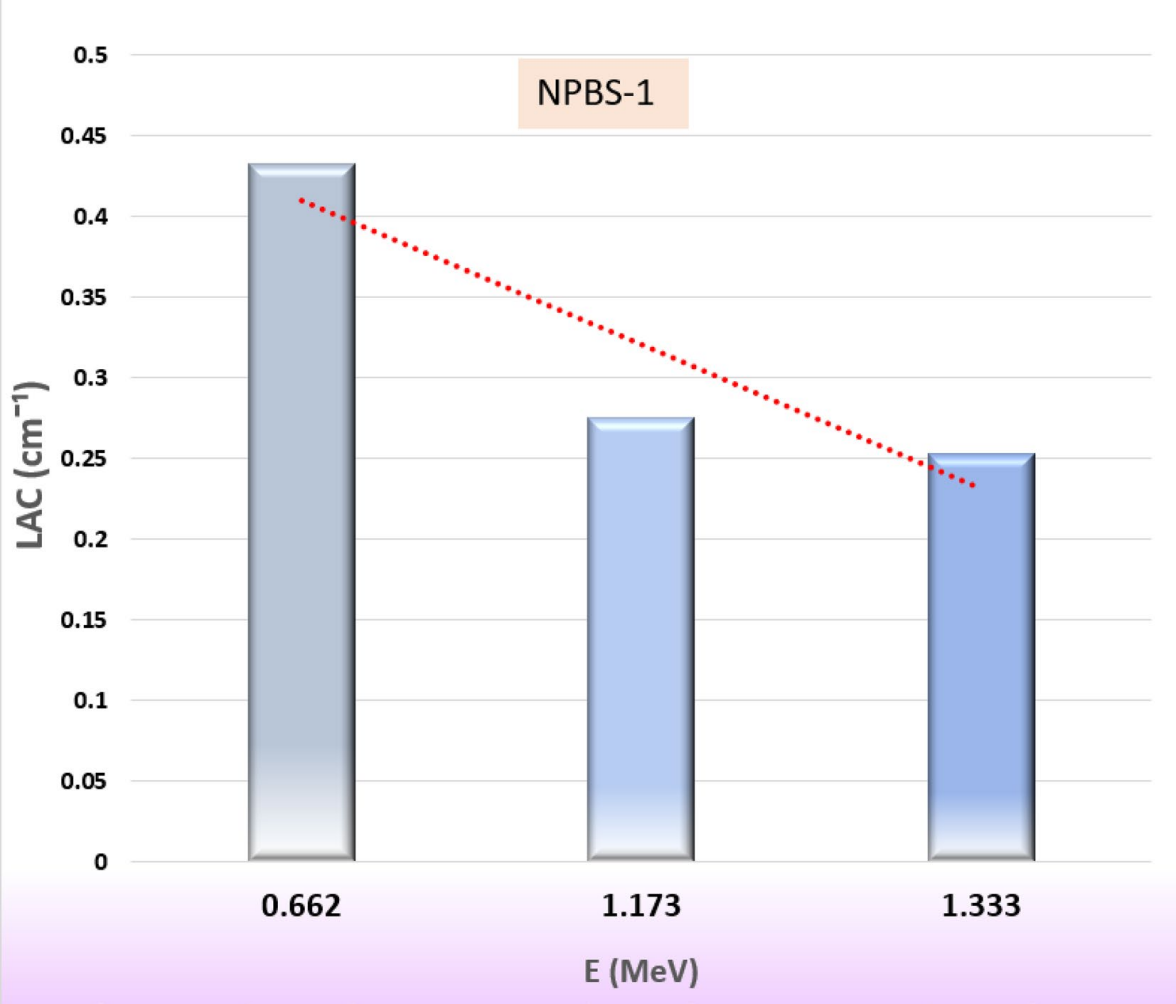
Influence of the gamma-ray energy (Eγ, MeV) on the linear attenuation coefficient (LAC, cm^− 1^), of the fabricated NPBS-1 glasses.

**Fig. 5:**
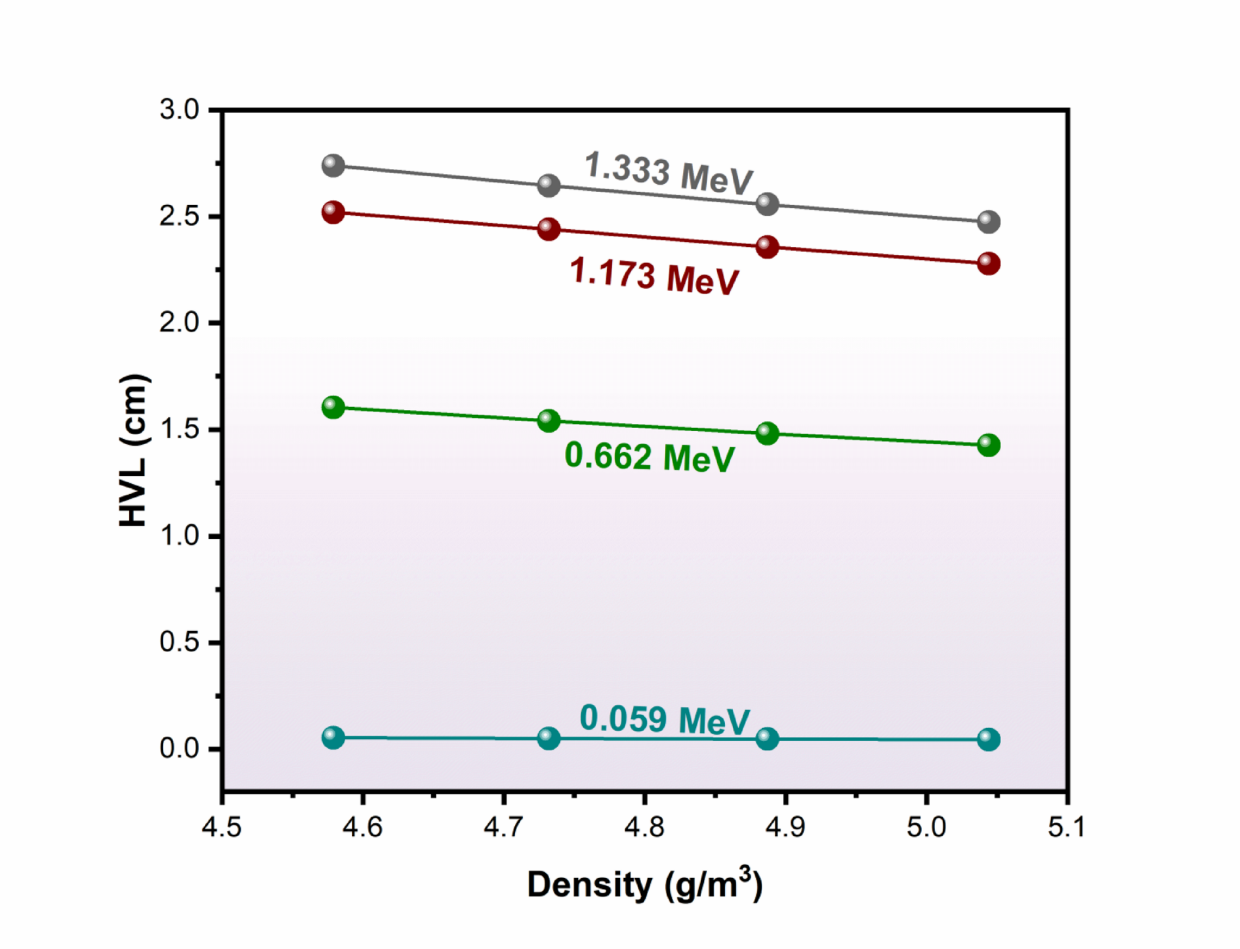
Influence of the density of NPBS glasses on the half value layer (HVL, cm), across the gamma ray energies.

**Fig. 6:**
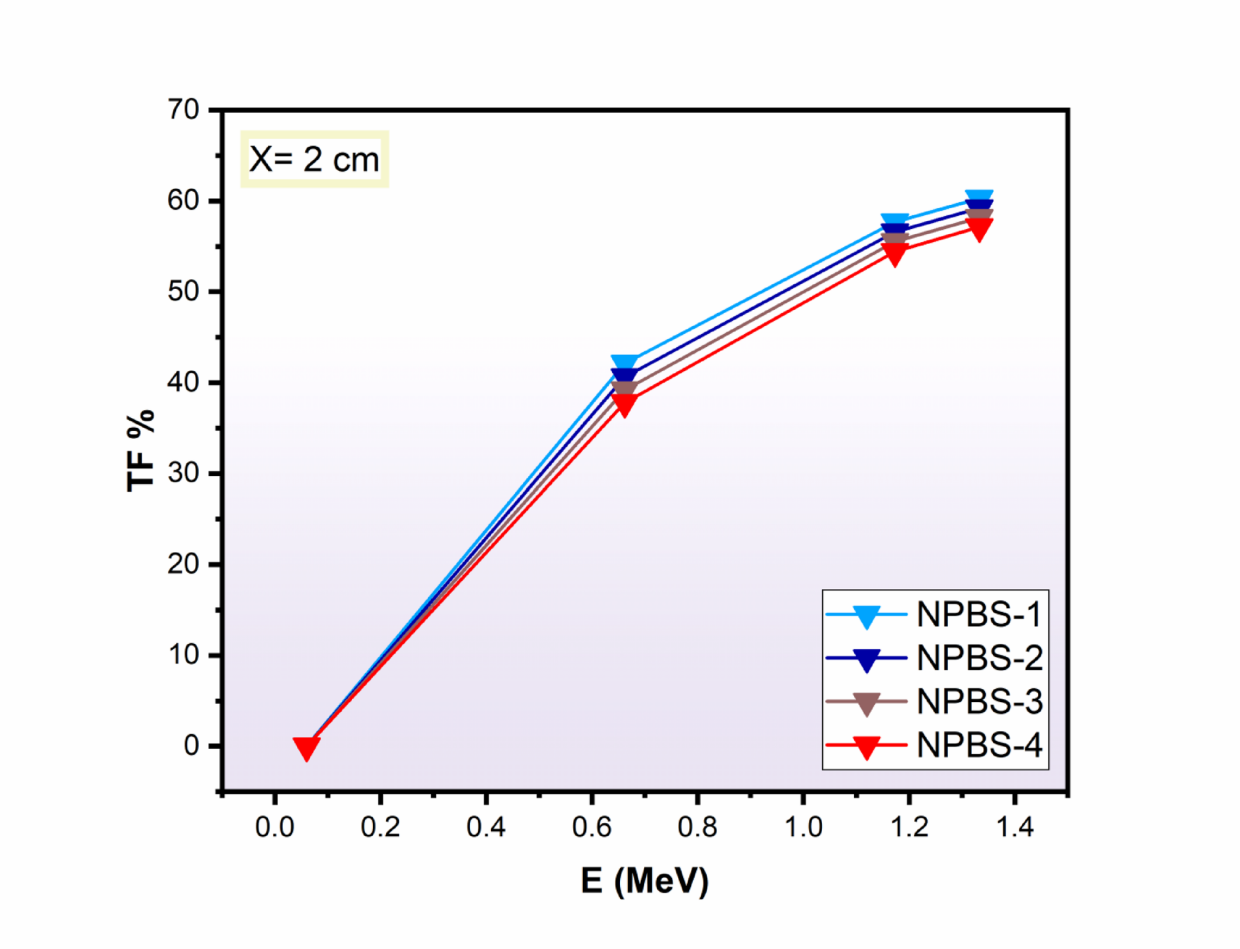
Influence of the γ-ray energy (Eγ, MeV) on the transmission factor (TF%), of the synthetic NPBS glasses.

**Fig. 7:**
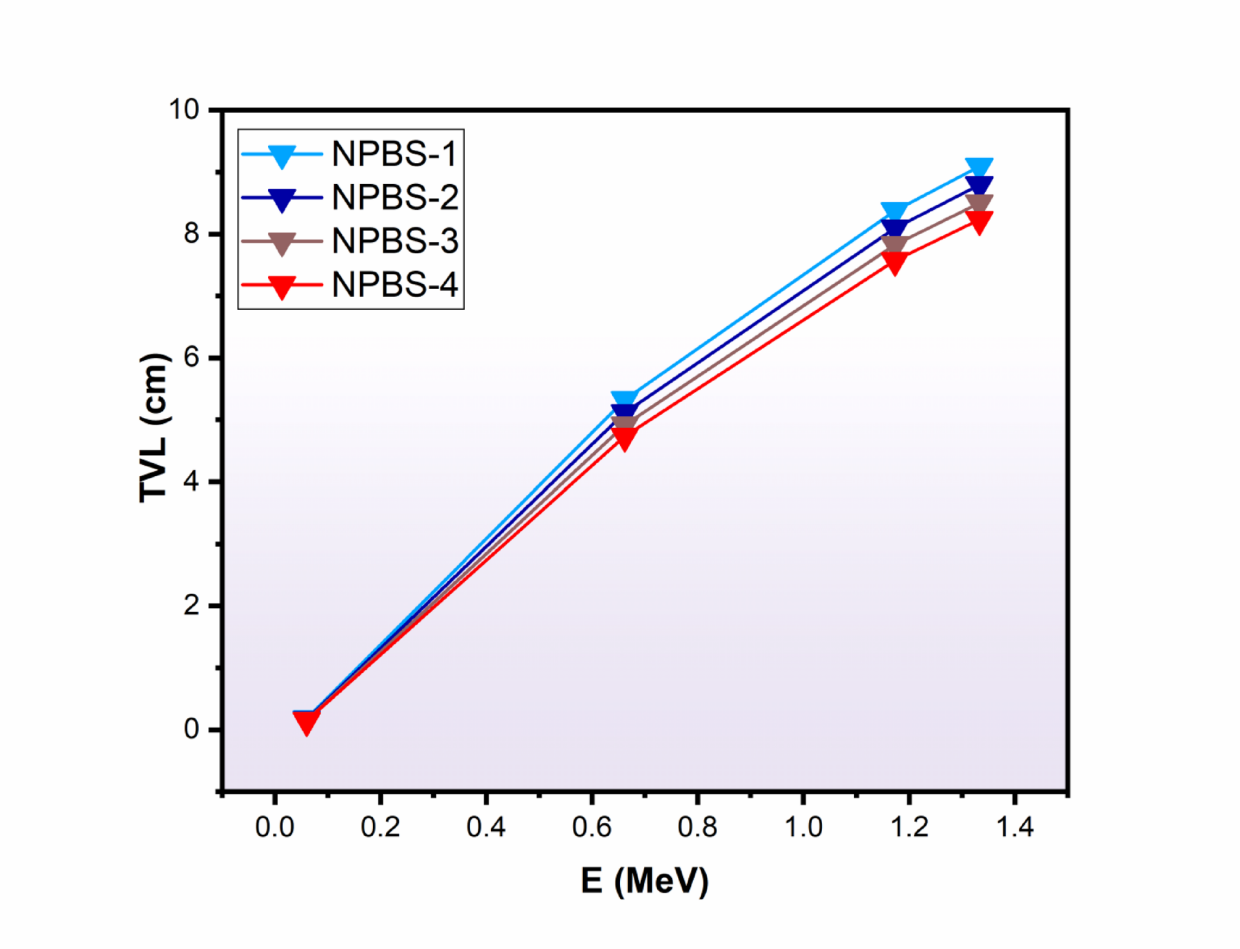
Influence of the γ-ray energy (Eγ, MeV) on the tenth value layer (TVL, cm), of the synthetic NPBS glasses.

Figures 4, 5 and 6, and 7 show the results of a study that determined several shielding parameters to determine the NBPS glasses’ ability to reduce incoming gamma rays. Figure 4 reveals the distinct trend of the LAC results as a function of energy (0.662, 1.173 and 1.333 MeV) and the NPBS glass composition. The LAC value of NPBS-1 dropped approximately 41% to 0.253 cm^− 1^ at gamma photon energy 1.333 MeV from 0.432 cm^− 1^ at 0.662 MeV. Furthermore, Fig. 4 illustrates the same trend of reduction that was observed for NPBS-2 (~ 42% decrease, 0.450 to 0.262 cm^− 1^), NBPS-3 (~ 42% decrease, 0.468 to 0.271 cm^− 1^) and NPBS-4 (~ 42% decrease, 0.486 to 0.280 cm^− 1^). The decreasing LAC values are affected mainly by the interactions between gamma photons and the NPBS glasses. At 0.662 MeV, the relatively higher value of LAC shows absorption of stronger gamma photon due to the photoelectric effect (PE) at the lower energies depending on the atomic number (Z). The progressive reduction of LAC values at 1.173 and 1.333 MeV, attributable to the dominance of Compton scattering (CS), a less Z- sensitive interaction mechanism and the cross section is proportional inversely with the gamma photon (σ_CS_ α ^−1^)^54,55^.

Moreover, the effect LAC with the NPBS chemical composition was studied and presented in Fig. 4. The LAC values increased progressively from NPBS-1 to NPBS-4 across all gamma photon energy levels. At 0.662 MeV, approximately 12.5% enhancement was detected when the LAC values increased from 0.432 cm^− 1^ (NPBS-1) to 0.486 cm^− 1^ (NPBS-4). This behavior suggests the NPBS-4 as superior radiation shielding efficiency at the low gamma energies due to the compositional differences like the higher density and the doping of heavy elements (Pb) compared to the NPBS-1.

The HVL of NPBS glasses was investigated across the applied gamma photon energies as a function of the density and presented in Fig. 5. The values of HVL exhibit a diminishing trend with the elevating NPBS glass density, confirming the glass composition with high density is a superior gamma shielding efficiency. According to Fig. 5, the higher value of HVL 0.053 cm (NPBS-1) is comparable to the lower value 0.044 cm (NPBS-4) which was detected at the low gamma photon energy 0.059 MeV, revealing the strong attenuation attributable to the photoelectric absorption. In contrast, the increasing gamma photon energy leads to an increase in the HVL values across all NPBS glass compositions. For example, the highest values of HVL 2.740, 2.646, 2.558 and 2.476 cm for NPBS-1 to NPBS-4, respectively, were measured at the gamma photon energy 1.333 MeV. Moreover, the remarked trend indicates that the doping lead oxide (PbO_2_) improves the radiation shielding features of the studied NPBS glasses at the lower and intermediate range of gamma energies. Figure 5 displays also the progressive trend of decreasing in HVL values with the variation of density from 4.579 g/cm^− 3^ (NPBS-1) to 5.044 g/cm^− 3^ (NPBS-4) proves the conclusion that NPBS-4 suggests that the most effective protection performance.

Figure 6 presents the variation of TF% at 2 cm thickness of four NPBS glasses with the increasing of gamma photon energy from 0.059 to 1.332 MeV. The results show the TF% diminishes as the NPBS glass’s density increase, confirming their enhanced attenuation capability. The TF values of NPBS glasses are nearly negligible at the low gamma energy 0.059 MeV, illustrating the strong photon absorption. Subsequently, the TF values range from 42.11 to 37.80% for NPBS-1 and NPBS-4, respectively, at 0.662 MeV, providing the increase of shielding efficiency with the increasing density. At higher gamma photon energies 1.173 and 1.333 MeV, the TF values remain relatively high due to the increased transmission of gamma photon through NPBS glasses. The TF values are 60.27% for NPBS-1 and reduced to 57.15% for NPBS-4, providing that although the lower effective of gamma photon attenuation at high gamma energies, the glass with the higher density still offer enhanced radiation shielding.

The TVL was also estimated for various applied gamma photon energies, as shown in Fig. 7. Like the same trend of HVL, the TVL values are reducing as the density of NPBS glasses increases from 4.579 to 5.044 g/cm^-3^. Figure 7 displays also the variation of TVL values with the increasing of the gamma photon energy from 0.059 to 1.333 MeV. At 0.059 MeV, the TVL values in changed from 0.177 cm for NPBS-1 to 0.146 cm for NPBS-4, indicating that the higher capability to attenuate the low gamma photon energies. At the intermediate gamma photon energy 0.662 MeV, the TVL values falls from 5.325 to 4.733 cm for NPBS-1 and NPBS-4, respectively, demonstrating the radiation shielding efficiency of the denser NPBS glasses. Consequently, The TVL values at the higher gamma photon energies such as 1.173 MeV and 1.332 MeV are significantly high owing to the provenance impact of the CS. The values of TVL at 1.333 MeV are 9.094 cm for NPBS-1 and reduced to 8.231 cm for NPBS-4, emphasizing the denser NPBS compositions provide better radiation shielding at high gamma energies. Therefore, the mentioned discussion reinforces the suitability of the NPBS glasses for the radiation shielding, especially in the applications requiring the capability to attenuate the high-energy gamma radiation.

**Fig. 8:**
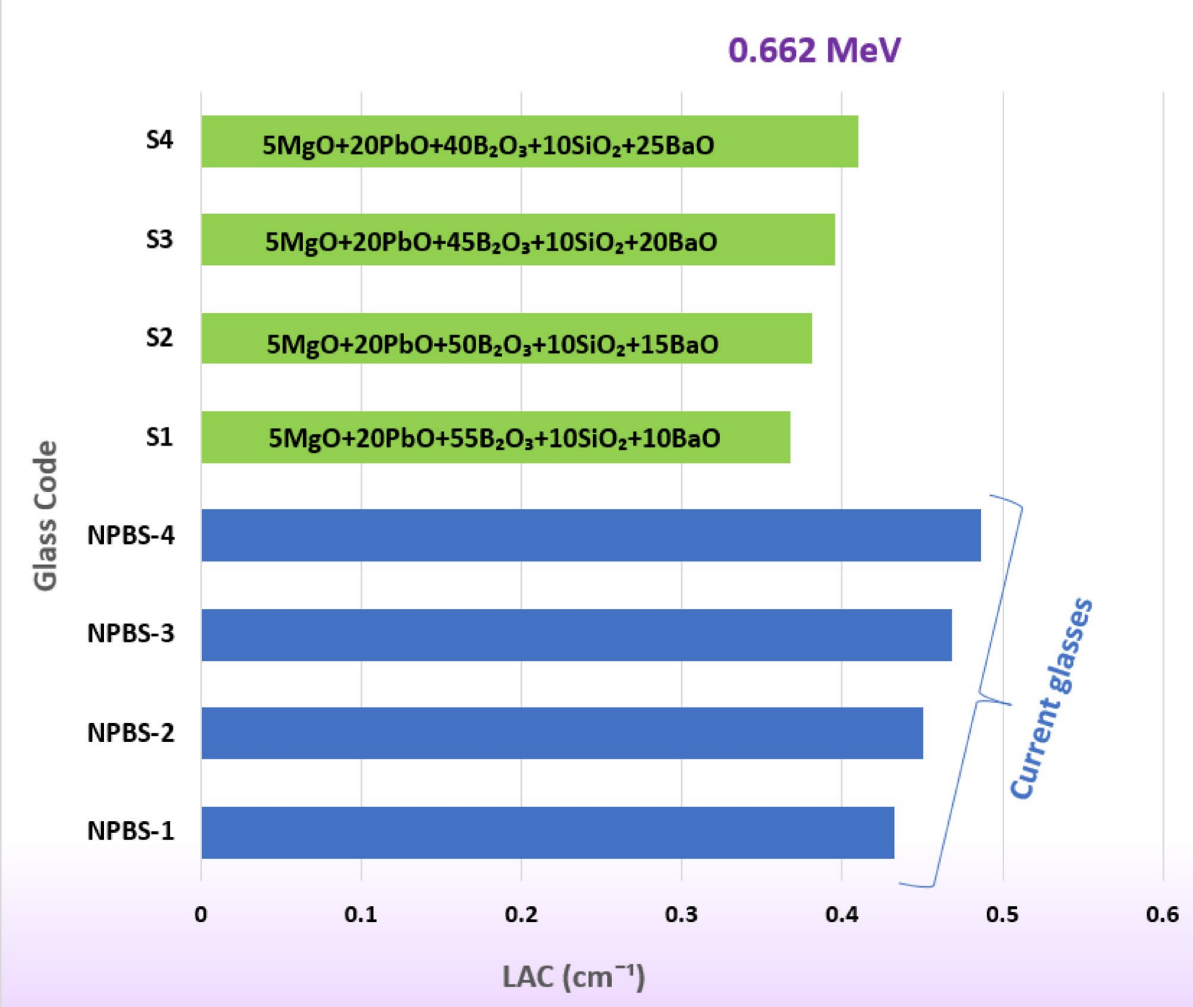
The comparison of the LAC of the NPBS glasses with the S glass series at gamma ray energy 0.662 MeV.

The LAC at 0.662 MeV was estimated for NPBS and to the other chemical composition of glasses including oxides such as MgO, PbO, B_2_O_3_, SiO_2_ and BaO and presented in Fig. 8^36^. As illustrated in Fig. 8, NPBS glasses reveals high values of LAC, with NPBS-4 having the higher attenuation capability among the present glasses NPBS and S series. The increasing of PbO in NPBS glass plays a fundamental role in improving the attenuation, as PbO has a high Z and effectively interacts with the gamma photons through the PE. The comparison with the glasses series S1-S4 containing BaO exposes generally the high values of LAC for NPBS glasses, showing the high performance of protection efficiency. The inclusion of BaO content in the glass S series leads to the enhancement of attenuation due to the high Z of Ba; however, the NPBS glasses including PbO remain outperform them.

## Conclusion

Glass samples with varying PbO_2_ concentrations (31, 33, 35 and 37 mol%) were prepared. The density of the glasses increases from 4.579 to 5.044 g/cm^3^ as a result of increase the PbO_2_ content. The radiation attenuation factors were experimentally determined at 0.059, 0.662, 1.173 and 1.333 MeV, using HPGe detector. The TF values of NPBS glasses are nearly negligible at the low gamma energy 0.059 MeV, illustrating the strong photon absorption. Subsequently, the TF values range from 42.11 to 37.80% for NPBS-1 and NPBS-4, respectively, at the intermediate gamma photon energy 0.662 MeV. At 1.333 MeV, the TF values are 60.27% for NPBS-1 and reduced to 57.15% for NPBS-4, providing that although the lower effective of gamma photon attenuation at high gamma energies, the glass with the higher density still offer enhanced radiation shielding. These materials have shown promising performance in reducing photon impact over a wide range of energy levels, making them suitable candidates for use in radiation shielding applications.

## Data Availability

The data that support the findings of this study are available from the corresponding author upon reasonable request.
